# Covalent-fragment screening identifies selective inhibitors of multiple Staphylococcus aureus serine hydrolases important for growth and biofilm formation

**DOI:** 10.21203/rs.3.rs-5494070/v1

**Published:** 2024-12-13

**Authors:** Matthew Bogyo, Tulsi Upadhyay, Emily Woods, Stephen Ahator, Kjersti Julin, Franco Faucher, Marijn Hollander, Nichole Pedowitz, Daniel Abegg, Isabella Hammond, Ifeanyichukwu Eke, Sijie Wang, Shiyu Chen, John Bennett, Jeyun Jo, Christian Lentz, Alex Adibekian, Matthias Fellner

**Affiliations:** Stanford University; Stanford University; Stanford University; The Artic University of Norway; The Artic University of Norway; Stanford University; Stanford University; Stanford University; The Scripps Research Insititute; Stanford University; Stanford University; Stanford University; Stanford University; Stanford University; Stanford University; UiT—The Arctic University of Norway; University of Illinois Chicago; University of Otago

## Abstract

*Staphylococcus aureus* is a leading cause of bacteria-associated mortality worldwide. This is largely because infection sites are often difficult to localize and the bacteria forms biofilms which are not effectively cleared using classical antibiotics. Therefore, there is a need for new tools to both image and treat *S. aureus* infections. We previously identified a group of *S. aureus* serine hydrolases known as fluorophosphonate-binding hydrolases (Fphs), which regulate aspects of virulence and lipid metabolism. However, because their structures are similar and their functions overlap, it remains challenging to distinguish the specific roles of individual members of this family. In this study, we applied a high-throughput screening approach using a library of covalent electrophiles to identify inhibitors for FphB, FphE, and FphH. We identified inhibitors that irreversibly bind to the active-site serine residue of each enzyme with high potency and selectivity without requiring extensive medicinal chemistry optimization. Structural and biochemical analysis identified novel binding modes for several of the inhibitors. Selective inhibitors of FphH impaired both bacterial growth and biofilm formation while Inhibitors of FphB and FphE had no impact on cell growth and only limited impact on biofilm formation. These results suggest that all three hydrolases likely play functional, but non-equivalent roles in biofilm formation and FphH is a potential target for development of therapeutics that have both antibiotic and anti-biofilm activity. Overall, we demonstrate that focused covalent fragment screening can be used to rapidly identify highly potent and selective electrophiles targeting bacterial serine hydrolases. This approach could be applied to other classes of lipid hydrolases in diverse pathogens or higher eukaryotes.

## Introduction

*Staphylococcus aureus* is a Gram-positive, coccus-shaped, pathogenic bacteria that causes a range of clinical manifestations including skin and soft tissue infection, pneumonia, osteomyelitis, endocarditis, and bacteremia^1,2^. The primary reservoir of these bacteria is human skin and mucous membranes, with 35–40% of the population colonized by S. *aureus* in the anterior nares^3^. This opportunistic pathogen is also highly prevalent in hospital environments. Toxin production and biofilm formation are associated with more severe infections. Biofilms can form on host tissues or indwelling medical devices and provide a significant survival advantage by increasing antibiotic resistance by 1500-fold^4,5^. In addition, biofilm formation enables the persistence of chronic infections and results in increased morbidity and mortality rates^6^. Therefore, early detection and inhibition of biofilms is essential for preventing and managing *S. aureus* infections.

A key step in improving both diagnosis and treatment of *S. aureus* infections is identifying new targets that are easily accessible to small molecules, unique to *S. aureus*, and that contribute to growth and maintenance of biofilms. Serine hydrolases are a promising class of enzymes to target for therapeutic, diagnostic, and imaging applications^7,8^. Despite being linked to critical processes such as virulence, biofilm formation, and host-pathogen interactions in many pathogenic organisms, the specific functional roles, expression patterns, and localization of many bacterial serine hydrolases remain poorly characterized. This is in part because serine hydrolases share a common α, β-hydrolase catalytic domain and often have overlapping functions that can compensate for one another. In these cases, genetic tools are difficult to use for functional studies because multiple targets must be knocked out simultaneously to observe a phenotype. As a result, there is a significant unmet need to identify selective inhibitors for members of this family that could be used not only to assign specific biological functions to specific serine hydrolases but also to determine their potential as therapeutic and imaging targets.

Our previous efforts to study serine hydrolases in *S. aureus* using a broad-spectrum activity-based probe, identified a family of serine hydrolases termed fluorophosphonate-binding hydrolases (Fphs; named sequentially FphA-J based on their predicted molecular weight)^9,10^. Of the few structurally and biochemically characterized Fphs, all belong to the α, β-hydrolase family and exhibit esterase or lipase-like properties. While FphB and FphE have been shown to have roles in *S. aureus* host-pathogen interactions^9^, their exact mechanism of action is unknown, making them important targets for further investigation. FphB is a 34 kDa membrane-associated enzyme that plays a role in establishing infections in the liver and heart^9^. It has homology with only a few Gram-positive bacteria and no homology with human proteins. FphE is a 31.2 kDa cytosolic serine hydrolase that lacks homologs in humans or other bacteria aside from *Listeria monocytogenes*^11^. FphH is a 30.1 kDa partially characterized cytosolic esterase thought to be involved in stress response, biofilm formation, and intercellular survival of *S. aureus*^12^. Despite this knowledge, the precise roles of these proteins in infection remain unclear.

FphE and FphH share a similar α, β-hydrolase domain but have unique substrate preferences. FphE prefers long chain (7–10 Carbon) fatty acid esters, while FphH prefers short chain (4 Carbon) fatty acid esters as substrates^11,12^. Transposon mutagenesis of the *fphH* gene results in an increase in levels of active FphE, suggesting a possible overlapping and compensatory function for these enzymes^13^. We have found that lipid-processing serine hydrolases in bacteria and other pathogens often have overlapping functions, making it difficult to observe phenotypes for some processes in single-gene mutants. Inhibiting compensatory enzymes of the same family simultaneously may help to uncover a general role for a family in growth and virulence^14,15^. Similarly, simultaneously targeting of Fph enzymes (FphB, FphE or FphH) with selective inhibitors could help uncover compensatory roles in growth, biofilm formation, or pathogenicity, which cannot be determined using a broad spectrum inhibitor of all Fphs.

Serine hydrolases process substrates using a hydrolysis mechanism in which an acyl-enzyme intermediate between the enzyme and the substrate is formed. Therefore, the catalytic nucleophile is a key target for serine-reactive electrophiles^8,16^. Fluorophosphonate-based electrophiles have been used to probe serine hydrolases in pathogenic bacteria^9,17^. In our previous work, we identified a highly FphB-selective chloroisocoumarin-based fluorescent probe by screening a 500-compound library designed to target serine proteases^9^. However, this class of electrophile was unstable under physiological conditions and therefore could not be used *in vivo*. We also identified triazole urea-based probes as selective inhibitors of FphE. However, identification of a selective lead molecule required significant medicinal chemistry efforts and focused only on a single class of electrophiles^13^. We also utilized a rational design strategy to optimize a promiscuous oxadiazolone-based electrophile warhead into a selective covalent inhibitor for FphE and demonstrated its utility for *S. aureus* infection imaging^11^. Inspired by these findings, we reasoned that performing a broad screen of diverse electrophile fragments could yield new classes of inhibitors and probes with high selectivity based predominantly on the electrophile.

Covalent inhibitors are becoming increasingly popular in drug discovery with approximately 30% of recently approved drugs being covalent^18^. Covalent inhibitors have several advantages over reversible binders as drug candidates and chemical probes. Notably, covalent inhibitors can be engineered to have exquisite selectivity and improved efficiency due to prolonged inhibition of the desired target^19,20^. They also have the potential to reduce dosing frequency and can reduce chances of inducing drug resistance due to the time dependent inhibition mechanism. The β-lactam antibiotics covalently target the active site serine of penicillin binding proteins yet only a limited number of covalent antibiotic classes have emerged in recent years^21^. This highlights the need for innovative approaches to find novel covalent inhibitors that can selectively target bacterial enzymes without off-target effects.

The most common approach used to discover covalent inhibitors is to incorporate an electrophile into an optimized reversible binding ligand, as reported for several FDA-approved kinase inhibitors^22^. While this strategy has proven effective, it is dependent on pre-existing reversible binders. To complement this approach, covalent virtual screening methodologies have also recently been used to identify new classes of electrophiles^23^. While successful, this *in silico* approach requires a high-resolution protein structure, cannot address protein flexibility, and cannot easily take the intrinsic reactivity of electrophiles into account. Another promising approach is covalent fragment screening, which focuses on low molecular weight (the range 200–500Da) compounds^24^ to identify potent hits directly during primary screening. Reports using covalent fragment screens mostly have focused on mammalian cysteine proteases or serine hydrolases involved in pathophysiological conditions. We hypothesized that screening a serine-reactive fragment library against bacterial serine hydrolases using a functional enzyme activity assay would help to identify new electrophile classes that could be used to generate inhibitors that are selective for each of the main Fph enzymes. This focused approach can streamline the subsequent hit-to-lead optimization process, potentially accelerating the development of covalent inhibitors for bacterial enzyme targets such as the Fph enzymes.

We describe here an enzyme inhibition screen of a library of 1,600 compounds, containing diverse electrophile fragments with preferential reactivity for serine nucleophiles. We chose to screen the library against three biologically important serine hydrolases in *S. aureus*: FphB, FphE, and FphH. The screen uses a small-volume, high-throughput compatible, highly sensitive enzyme assay that significantly reduced consumption of assay components and inhibitors. Primary screening identified both potent and highly selective lead molecules, including multiple electrophile classes that have not previously been reported to target the active site of a serine hydrolase. Further testing of these inhibitors confirmed both their activity and selectivity against the native Fph targets in live bacteria. X-ray crystallography validated the binding mode of several of the top electrophiles, revealing key residues involved in active site binding and providing a foundation to enable improvement of the initial hit compounds. Finally, the identified selective inhibitors enabled studies in live bacteria to define the function of the Fph enzymes in bacterial growth and biofilm formation.

## Results

### Serine-based covalent fragment library screening

**Figure 1:**
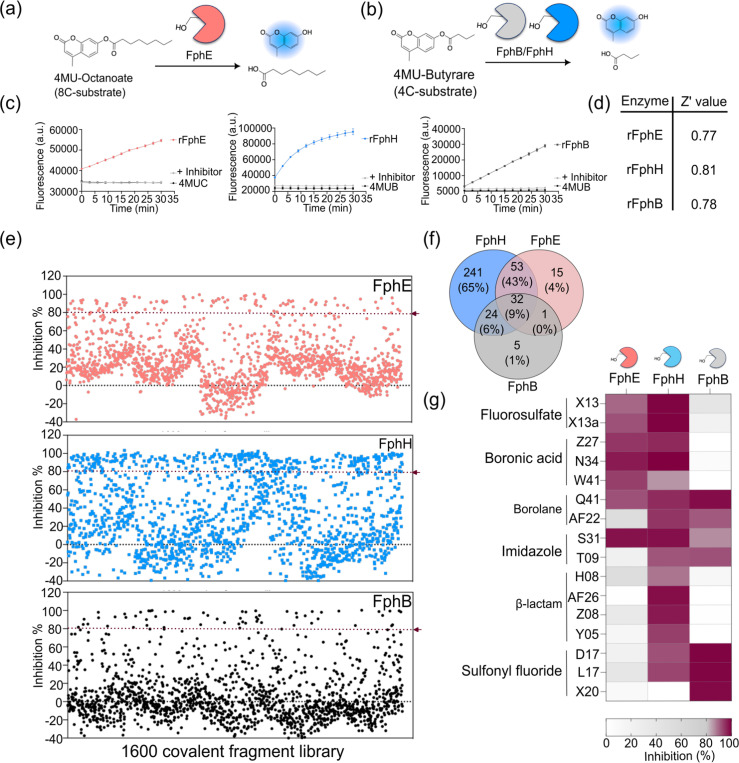
High-throughput screening against *S. aureus* serine hydrolases. (a-b) Schematic representation of the enzyme assay used in the high-throughput screening of purified rFphE, rFphH and rFphB using the substrates 4-methylumbelliferyl octanoate for FphE and 4-methylumbelliferyl butyrate for FphB and FphH. (c) Progress curves for each serine hydrolase (0.5 nM rFphE, 5 nM rFphH and 100 nM rFphB) using 20 μM of the substrate for each assay. (d) Calculated Z’ values (*n* = 8, mean ± SD) for each enzyme. (e) Plot of the percent inhibition values (at 50 μM) for each compound in the primary screening fragment library for FphE (red), FphH (blue) and FphB (black). An FP-based inhibitor was used as a positive control. (f) Venn diagram showing the distribution of the top hits with ≥80% inhibition among the three targets. (g) Heat maps for the top hits arranged according to their electrophile type and percent inhibition for each target enzyme.

To establish a setup for identifying selective inhibitors for each target Fph enzyme, we developed a high-throughput screen (HTS) using a fluorescent-based enzyme assay. For each of the three recombinantly expressed and purified Fphs, we used an optimal fluorogenic lipid ester. In our previous reports, we have used a 4-methylumbelliferone (4MU) substrate library consisting of lipid esters ranging from chain length of C2 to C10 to define the substrate preference for each of these Fph enzymes^9,11,12^. Both FphB and FphH prefer to cleave lipid esters with 4 carbon atoms and therefore effectively process the 4-methylumbelliferyl butyrate (4MUB) substrate, while FphE prefers 7–10 carbon atoms and preferentially cleaves the 4-methylumbelliferyl caprylate (4MUC) substrate (Fig. 1a, b). We optimized the buffer conditions and enzyme and substrate concentrations to generate a robust signal-to-noise ratio and suitable Z’ values^25^ (Fig. 1c). We used a fluorophosphonate-based (FP) inhibitor as a positive control^17^ and measured the highest enzyme activity with 50 μM fluorogenic substrates for recombinant FphB (rFphB) at a concentration of 100 nM, at 0.5 nM for recombinant FphE (rFphE) and at 5 nM for recombinant FphH (rFphH). The observed Z’ in the assays for each protein was > 0.7, indicating sufficient signal-to-noise for reliable hit identification (Fig. 1d). To establish the assay conditions for the 384-well HTS format, we scaled down the reaction volume to 10 μL to minimize the amount of inhibitor, enzyme, and substrate needed for each screen. To dispense the library of fragments we used an Echo acoustic liquid handler for precise nanoliter dispensing to a 384-well screening plate. This technique is fully automated, rapid, highly accurate and scalable, with the dispense volume ranging from 2.5 nL to 1 μL. We used the optimized assay conditions for each Fph to screen a serine-focused covalent fragment library of 1,600 commercially available compounds. The library is composed of boronic acids or borolanes (21.7%), sulfonyl fluorides (SF, 14.6%), fluorosulfates (OSF, 9.8%), β-lactams (6.5%), carbaldehydes (20.3%), imidazoles (2%) and other electrophiles (urea, indole, oxirane etc., 25.1%; Table S1).

For our primary screens we used a final concentration of each fragment of 50 μM such that the final concentration of DMSO in the assays was 0.5% v/v. Compounds were preincubated for one hour prior to addition of the substrates. To normalize the screening data, we used 50 μM of a Fluorophosphonate-based (FP) inhibitor as a positive control (Fig. 1e). Using the inhibition threshold of ≥ 80%, we identified a total of 371 active hits across the three enzymes. Of these, we found a total of 350 hits for FphH, 101 hits for FphE and 62 hits for FphB, respectively (Fig. 1f, Table S1). The overall distribution of inhibitors among the three Fphs shows that 9% of the hits are common for all three hydrolases.

We were pleased to see that hits could be identified for each of the three Fph targets that showed potentially selective inhibition. Interestingly, FphH had a substantially higher number of total hits (both selective and not selective) compared to the other two targets, suggesting that it may be more accessible to inhibition by a wider range of covalent electrophiles. We observed a similar trend in the structure-activity landscape index (SALI) plots determined by DataWarrior tool^26^ using the OrgFunction descriptor and inhibition percentage for each enzyme as an identifier. This analysis clustered the compounds based on the common functional groups with similar inhibition activity (Figure S1a–c).

### Identification of novel electrophiles targeting the S. aureus serine hydrolases

**Figure 2:**
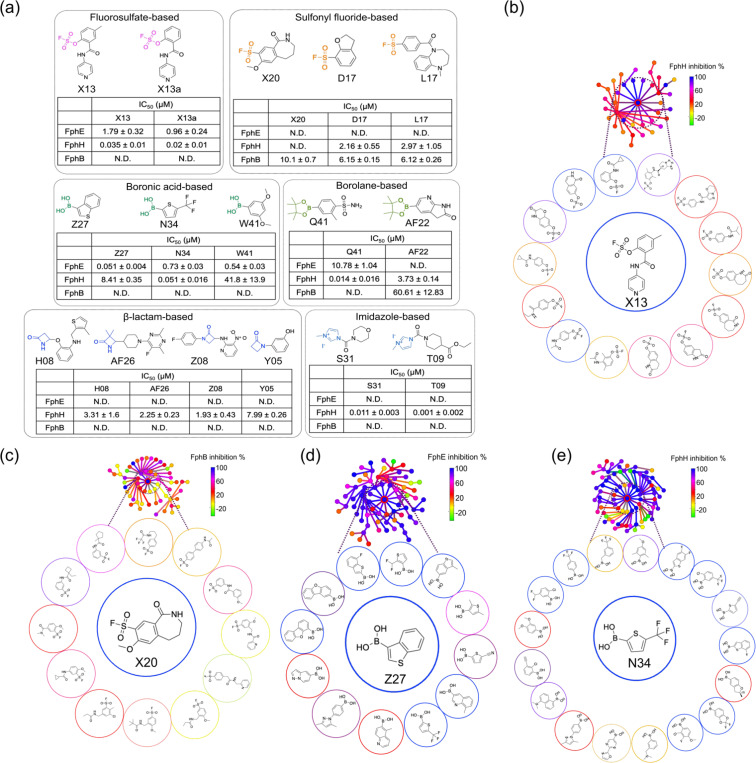
Identification of multiple electrophile-classes for *S. aureus* serine hydrolases (a) Structures of hit compounds, selected based on their selectivity against each Fph enzyme, organized by electrophile-class. The measured IC_50_ values for each target are shown for each set of hits. N.D. indicates that the IC_50_ value could not be determined due to incomplete inhibition at the highest concentration tested. (b-e) Generation of structure activity relationship data using the neighborhood tree generated from the SALI plot (shown in Figure S1b–d). Plots show closely related structures based on the OrgFunction descriptor for each electrophile and their corresponding activities for each target Fph enzyme.

After grouping the electrophiles from the initial set of over 300 hits for all three Fph targets, we selected the top 16 compounds based on potency and potential selectivity (Fig. 1g). We also selected hits that covered a diverse range of electrophiles with > 90% inhibition of activity for each enzyme for further analysis. Moreover, similar backbone structures in the library provided structure-activity relationship (SAR) data for the top hits (Fig. 2b–e, Figure S1). These criteria helped us to select fragments which could be directly used as inhibitors with minimal synthetic modifications. To test the potency of the top hits obtained from the primary screening for each target, we used the Echo dispensing system to perform a preliminary 4-point dose response (0.8, 10, 25 and 50 μM) using 2.5 to 50 nL inhibitor volumes. This allowed us to determine a crude IC_50_ value for each hit. Surprisingly, we found some of the hits had IC_50_ values in the nanomolar range, giving additional confidence to further explore these electrophiles. To further validate the inhibition potency and selectivity for the selected hits against each Fph enzyme, we determined the dose-dependent effect of all 16 hits against the three Fphs separately (Table S2, Of the top hits selected, we focused on compound X13 because it contains a fluorosulfate (OSF) electrophile which has recently gained attention due to its overall low reactivity, high stability and ability to target multiple amino acids including histidine, lysine and tyrosine^27^. We were intrigued by this hit as the OSF electrophile has never been shown to covalently target a serine residue and we therefore wanted to determine if this hit is functioning by direct binding to the active site serine. The X13 fragment was active against rFphH and rFphE, with IC_50_ values in the nanomolar range for rFphH (Fig. 2a, Table S2, Figure S2). Our SAR analysis using the DataWarrior tool also showed that X13 is a not a promiscuous electrophile and has the highest activity for rFphH (Fig. 2b, Figure S1d). The second set of electrophiles we chose to focus on were sulfonyl fluoride (SF)-based fragments (X20, L17, D17). The SF hit X20 is the only fragment that we identified that was selective for rFphB (Fig. 2a). DataWarrior analysis also showed that no related structures have inhibition activity against rFphB (Fig. 2c, Figure S1e). The third class of electrophiles that we selected for follow up studies were boronic acid-based fragments. Given the high potency of this class of electrophiles for serine nucleophiles we were not surprised to see that 84% of the total boronic acid compounds in the library inhibited rFphE and rFphH at the initial high concentration used for screening (Table S1), which was further confirmed in the DataWarrior analysis (Fig. 2d–e, Figure S1 f–g). However, we selected a set of three hits (Z27, N34, and W41) because of their high potency and selectivity for FphE and FphH (Fig. 2a). Specifically, our dose response results indicated that Z27 had a 165-fold selectivity index for rFphE over rFphH, whereas N34 had a 14-fold selectivity for rFphH over rFphE. These results suggest that both N34 and Z27 are highly potent inhibitors that may have sufficient selectivity to allow them to be used for functional studies of each of these two important Fph enzymes. Additionally, we found that borolane-based inhibitors Q41 and AF22, that showed inhibition of all three Fphs in primary screening, were quite selective for rFphH in the dose-dependent response assay (Fig. 2a). Lastly, our top hits also included β-lactam-based (Y05, AF26, H08, Z08) and imidazole-based (T09, S31) fragments. All the β-lactam hits showed selectivity for rFphH with single-digit micromolar IC_50_ values (Fig. 2a). The imidazole-based inhibitor, S31 showed inhibition of rFphH and rFphB, and T09 was highly potent against rFphH with IC_50_ = 1nM in the enzyme inhibition assay (Fig. 2a, Table S2, Figure S2). Despite initial promise with both β-lactam and imidazole-based inhibitors, none of the hits showed any covalent labeling by mass spectrometry or crystallographic analysis and did not show competition in labeling of the recombinant rFphH (Figure S3).

### Fluorosulfate (OSF)-based inhibitors as potent covalent inhibitors of FphH

Fluorosulfates are highly stable but weak electrophiles. They can undergo sulfur (VI) fluoride exchange (SuFEx) reactions, which occurs when a nucleophile attacks the sulfur center and displaces the fluoride ion^28,29^. These electrophiles primarily react with tyrosine, lysine, and histidine residues and have been used to target non-enzymatic proteins through disruption of protein-protein interactions (PPIs)^27^. A few examples also suggest their use in inhibiting enzyme activity by targeting Tyr or Lys in the active site^30^. However, there are currently no examples of ligands containing the OSF electrophile that target catalytically active serine residues. Therefore, we were surprised to find the OSF as a hit in our enzyme activity screens.

In the first round of primary screening, from a total of 157 OSF-based fragments in the library, we obtained 29 hits inhibiting FphH, whereas only a few of these compounds inhibited rFphE and rFphB. In the second round of 4-point dose response assays performed on the 29 hits, we found X13 to be the only hit showing 100% inhibition of both rFphE and rFphH activity in the sub-micromolar range. For further studies, we re-synthesized X13 and confirmed purity by NMR and LC/MS (SI details). In secondary screening, we assessed the inhibition potency of the lead compounds for the recombinant enzymes over a concentration range from 100 nM to 50 μM. X13 showed dose-dependent inhibition of both rFphE and rFphH, with nearly 100 times higher potency for rFphH (IC_50_ = 0.035 μM) compared to rFphE (IC_50_ = 1.79 μM), and no activity against rFphB (Fig. 2a, Figure S2b).

To confirm that inhibition was due to the OSF-electrophile on the X13 fragments, we made an analog that lacked the reactive fluorine (Figure S4a). This control molecule did not inhibit the activity of rFphH (Figure S4b). A limited SAR study also showed that while the methyl group on the aromatic ring was not important for potency, the pyridine ring is essential for activity, and removing the nitrogen from the ring resulted in loss of X13 inhibition potency (Figure S4b, S4c). With this information, we synthesized an analog of X13 (X13a) which lacks the methyl substitution on the phenyl ring, resulting in overall improved inhibition potency for both rFphH (IC_50_ = 0.027 μM) and FphE (IC_50_ = 0.96 μM; Fig. 2a).

**Figure 3:**
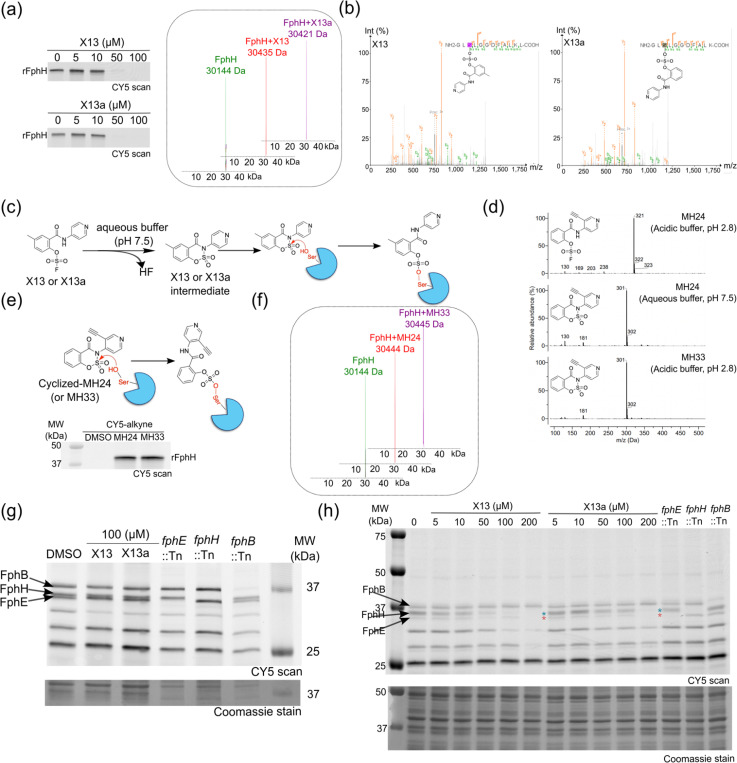
Biochemical characterization of fluorosulfate-based fragments. (a) Fluorescent SDS-PAGE gel images showing concentration-dependent inhibition of the labeling of rFphH (500 nM) by FP-Cy5 probe (300 nM) for X13 and X13a. Deconvoluted intact protein mass spectra of 1μM rFphH alone or after 1hr incubation with 10 μM X13 or X13a. (b) MS/MS spectra confirming covalent modification of the active site Ser113 of rFphH by X13 and X13a. (c) Proposed mechanism of action of compounds X13 and X13a involving initial cyclization to eliminate HF, followed by ring opening by the active site serine. (d) LC/MS profiles for X13d and X13e in LC/MS buffer or aqueous buffer. (e) Proposed reaction mechanism of X13d or X13e binding to rFphH, Fluorescence SDS-PAGE image of rFphH for direct visualization of labelling by clickable probes X13d and X13e with Cy5-azide. (f) Native mass spectrometry (MS) characterization of 1μM rFphH alone or after 1hr incubation with 10 μM X13d or X13e. (g) Competition of FP-Cy5 labeling of live *S. aureus* cells (USA300 LAC) or the individual transposon mutants for f*phE, fphH* and *fphB* after preincubated with 100 μM X13 and X13a for 120 min, incubated with clickable 1μM FP, lysis and labeling with Cy5-azide and analysis by SDS-PAGE and fluorescence scanning. (h) Competition of FP-Cy5 labeling of *S. aureus* lysates. *S. aureus* USA300 WT cells were lysed prior to preincubation with the indicated doses of X13 and X13a for 120 min, labelled with clickable FP-Cy5, and analyzed by SDS-PAGE and fluorescence scan. The indicated Fph transposon mutants (::Tn) lysates are included to highlight the location of the three enzymes in the gel. All data shown are representative of three independent experiments.

To understand whether X13 and X13a bind covalently to the target enzymes through a specific covalent reaction, we first performed active site labeling of rFphH. We used a broad spectrum active site label of serine hydrolases, FP-Cy5, in the presence of X13 and X13a and found dose-dependent competition of protein labeling, further confirming that both are binding to the active site serine of rFphH (Fig. 3a). Furthermore, incubation of rFphH with X13 or X13a in a 1:10 ratio for one hour resulted in a single mass shift of the intact protein of 291 Da for X13 and 308 Da for X13a, confirming covalent binding at a single site (Fig. 3a). To assess whether X13 and X13a bind to the active site serine, we performed tandem mass spectrometry analysis and confirmed a single modification corresponding to the covalent attachment of each fragment to the active serine (Fig. 3b). No labeling of rFphE by X13 was observed by mass-spectrometry, suggesting a unique mechanism of binding with rFphH.

To measure the stability of these electrophiles in aqueous buffer, we incubated X13 and X13a in aqueous assay buffer (1X Phosphate buffer saline) at pH 7.5 for 60 min and then measured the resulting LC/MS spectra. Surprisingly, we observed a mass shift of 20 Da upon incubation of the compounds when added to aqueous pH 7.5 buffer even in the absence of protein. This shift was not observed in the acidic mass spectrometry buffer (0.1% formic acid (FA) in H_2_O as buffer A, and 0.1% FA in acetonitrile (ACN) as buffer B; SI details). We therefore reasoned that the molecule likely reacts to release the fluoride under neutral pH conditions (Fig. 3c). Given the presence of the amide nitrogen in close proximity to the sulfur (VI) atom we reasoned that it likely produces a saccharine-like structure by a cyclization to release the fluorine atom^31^. This would create a molecule that could then be ring-opened by attack by the active site serine on the sulfur center, resulting in irreversible protein modification. To confirm the identity of the active intermediate, we synthesized an alkyne version of X13a for direct protein labeling studies. We were able to purify the non-cyclized OSF-fragment, X13d under acidic conditions, as well as the cyclized OSF-fragment, X13e. We observed a clear mass difference of 20 Da between the two purified species by LC/MS analysis. Furthermore, the non-cyclized version was rapidly converted to the cyclic version upon incubation in pH 7.5 buffer, consistent with our proposed mechanism (Fig. 3d). Both the X13d and X13e probes showed similar labeling intensity of rFphH (Fig. 3e) and both produced the expected mass shift of the protein by mass spectrometry confirming our hypothesis that the cyclized intermediate is the active species that reacts with FphH (Fig. 3f).

To determine the binding specificity of X13 and X13a, we incubated live bacteria with 100 μM of each compound followed by labeling with FP-alkyne. Cells were then lysed and click labeled with sulfo-Cy5 azide and analyzed by SDS-PAGE. Neither of the compounds competed with FP labeling in intact cells (Fig. 3g). We hypothesized this lack of competition may have been due to the inability to permeate the cell membranes. For bacteria, the permeability is influenced by the polarity of the compound, measured by clogP. Drugs targeting Gram-negative bacteria show higher polarities (clogP = − 0.1) compared to those targeting Gram-positive bacteria (clogP = 2.1)^32,33^. The clogP values for cyclized X13 and X13a are 1.22 and 0.724, respectively, suggesting that polarity is limiting the permeability of the compounds. To test this hypothesis, we incubated X13 and X13a with *S. aureus* lysates. Lysate labeling showed that both the compounds dose-dependently competed with FP labeling of rFphH as well as rFphE at higher concentrations (Fig. 3h). Overall, this electrophile has a unique mechanism of binding with the active site serine of FphH and may be a valuable new electrophile class to target other serine hydrolases.

### Sulfonyl fluorides-based irreversible warhead against FphB

Another potentially valuable electrophile type we identified in our screening was the sulfonyl fluoride (SF). These electrophiles have relatively high reactivity but show a preference for Ser > Thr > Tyr > Cys > Lys > His^16^. The SF electrophile has been widely used due to its unique balance between reactivity and stability under physiological conditions. Although SF electrophiles can modify several amino acids, they can be designed to react only when bound in an active site of an enzyme. Several SF-based inhibitors have been reported for serine hydrolases or lipases in which the electrophile is attached to saturated carbon chains^34^. Of the 233 SF fragments in the screening library, we found 104 hits in the 80% cutoff range for FphH. To focus only on the most potent of these molecules we increased the cut-off to 95% inhibition for FphH, which reduced our hit count to 33. In the 4-point dose-response assay, only five hits demonstrated dose-dependent inhibition with IC_50_ values less than 15 μM for FphH. However, most of the hits that demonstrated 80–95% inhibition were non-selective and inhibited both FphE and FphB.

**Figure 4:**
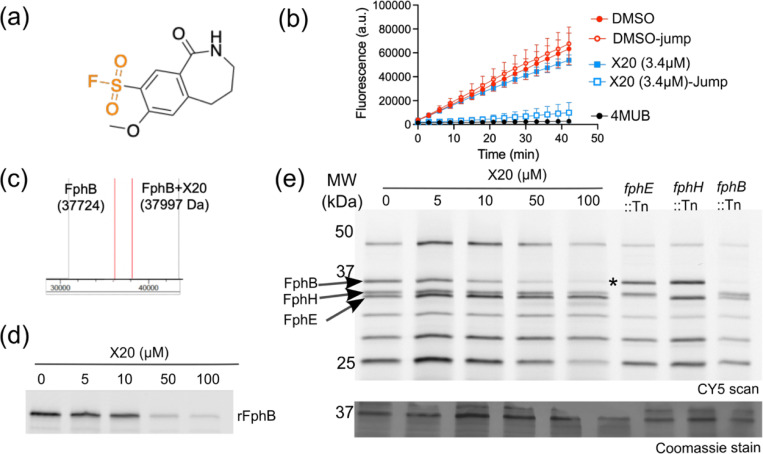
A sulfonyl fluoride-based irreversible electrophile is selective for FphB. (a) The structure of FphB-selective hit compound, X20. (b) Jump dilution assay of rFphB inhibited by X20. rFphB was incubated for 60 min with 101 μM (10X IC_50_) of X20, followed by a 30-fold dilution prior to performing enzyme activity assay. Activity from this jump-dilution was compared with a DMSO control and 3.75 μM of X20. (c) Deconvoluted mass spectra of 1μM rFphB alone or after 1hr incubation with 10 μM X20. (d) Fluorescent SDS-PAGE gel image showing the competition of FP-Cy5 labelling of rFphB preincubated with the indicated doses of X20 and subsequently labelled with the FP-Cy5 probe (500 nM). (e) Competition of FP-Cy5 labeling of live *S. aureus* cells. USA300 WT cells were preincubated with the indicated concentrations of X20 for 120 min. The individual transposon mutants of f*phB, fphH and fphE* are included to highlight the location of the three enzymes in the gel. The cells were incubated with 1μM FP-Alkyne, lysed, labelled with Cy5-Azide, analyzed by SDS-PAGE and analyzed by fluorescence scan. All data shown are representative of three independent experiments.

For FphB, we identified only 14 hits (with > 80% inhibition cut-off), and five compounds demonstrated dose-dependent inhibition. Of these, X20 was the only hit from any of the electrophile classes that showed selectivity against rFphB (IC_50_ = 10.1 μM). The closely related compounds, D17 and L17, showed similar inhibition against rFphB (IC_50_: D17 = 6.15 μM and L17 = 6.12 μM), but also had activity against rFphH (IC_50_: D17 = 2.5 μM and L17 = 3 μM; Fig. 2a, Figure S2a). To determine if inhibition of FphB by X20 was covalent and irreversible, we performed a jump-dilution assay, where we incubated rFphB with X20 at concentration of 101 μM (10X IC_50_) to saturate binding of the active site, followed by a 30-fold dilution and then addition of the fluorogenic substrate (Fig. 4b). These data demonstrated that FphB activity remained inhibited after dilution, confirming irreversible binding of X20 to rFphB. As a further confirmation of direct covalent binding of X20 to rFphB, we performed mass spectrometry using a mixture of rFphB and X20 in a 1:10 ratio (Fig. 4c). This result showed that a single X20 adduct formed, presumably due to covalent labeling of the active site serine. Finally, we observed that X20 dose-dependently blocked labeling of rFphB by the general serine hydrolase active site probe FP-Cy5, confirming that the compound binds in the active site (Fig. 4d).

Once we confirmed direct covalent modification of the active serine of rFphB, we performed competition labeling analysis using the FP-alkyne probe in live *S. aureus* cells. We incubated cells with increasing concentrations of X20 prior to labeling with FP-alkyne, followed by cell lysis and click labeling with sulfo-Cy5 azide and SDS-PAGE to measure the residual activity of each of the Fph proteins. This analysis confirmed that X20 retains both its potency and selectivity towards the natively expressed FphB in the cell envelope of live *S. aureus* cells and suggests that it will be a valuable new tool for studies of FphB function (Fig. 4e).

### Boronic acid-based fragments have nanomolar potency and high selectivity for FphE and FphH

**Figure 5:**
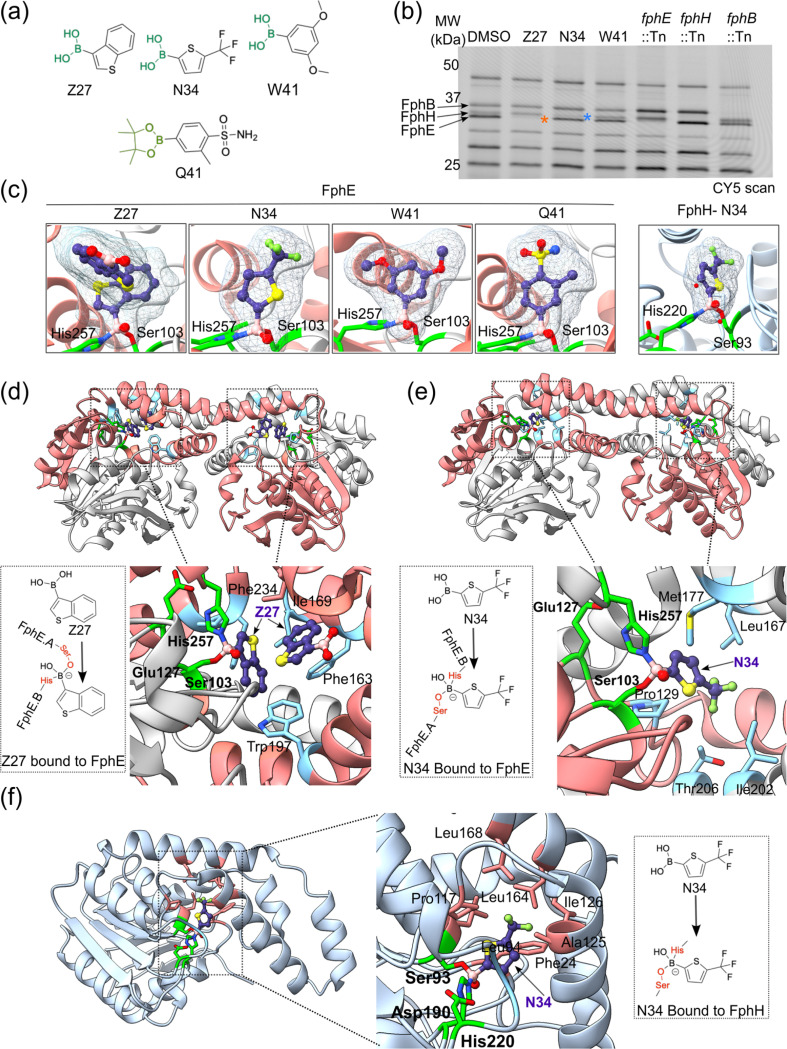
Boron based inhibitors of FphE and FphH. (a) The structure of the three top boronic acid hits, as well as one of the promiscuous borolane hits. (b) Fluorescent SDS-PAGE gel image showing competition of FP labeling in intact *S. aureus* cells by 100 μM Z27, N34 and W41. The individual transposon mutants of *fphB, fphH* and *fphE* are included to highlight the location of the three enzymes in the gel. (c) Close-up view of FphE covalently inhibited by Z27 (also includes an unreacted non covalently bound molecule; PDB ID 8UGM, 1.65 Å), N34 (8TFW, 1.93 Å), W41 (8UIX, 2.39 Å) and Q41 (8UWM, 1.97 Å). Boron binds to the active site FphE-serine and histidine from different homodimer FphE monomers. FphH binds N34 using the active site serine and histidine residues from the same monomer (8TAV, 1.39 Å). Ligand bound to FphE-Ser103 and FphE-His257 or FphH-Ser93-His220 are shown as a blue mesh. (d) Structure of FphE bound by Z27 showing the dual attack mechanism using serine and histidine. The structure also shows a second unreacted bound Z27 molecule in the active site that does not engage the catalytic residues. (e) Structure of FphE bound by N34 showing the dual attack mechanism using serine and histidine. (f) Structure of FphH bound by N34 through a di-covalent interaction with the active site serine and histidine.

The third class of electrophile fragments that we selected as hits from the initial screen contained boronic acids. These compounds are mild electrophiles that form covalent reversible adducts to serine, making them potentially valuable as therapeutic agents. Of the 85 boronic acid-based fragments in the library, 50% of them inhibited both FphE and FphH, and three hits inhibited FphB. This encouraged us to select Z27, N34, and W41 as the three top hits, since these molecules showed inhibition potency in the nanomolar range and substantial selectivity for either FphE or FphH (Fig. 5a). Specifically, the fragment Z27 was both highly potent for rFphE (IC_50_ = 50 nM) but also highly selective with more than 100-fold lower potency for rFphH (IC_50_ = 8.38 μM). In addition, the fragment N34 had high potency for rFphH (IC_50_ = 60 nM) with a 12.5-fold reduction in potency for rFphE (IC_50_ = 0.73 μM). The fragment W41 was slightly less potent than N34 for rFphE (IC_50_ = 0.54 μM) but had nearly 100-fold selectivity over rFphH (IC_50_ = 49.7 μM). None of the molecules showed any inhibition of rFphB (Fig. 2b, Figure S2). Further, we observed that N34 dose-dependently blocked labeling of rFphH by the active site probe FP-Cy5, however Z27 inhibited labeling of both rFphE and rFphH at > 50 μM. The data suggests that these two compounds bind in the active site of the enzymes and N34 is a more potent inhibitor of rFphH (Figure S5a).

To confirm that the specificity of the three hits for the purified enzymes was retained for the native *S. aureus* targets, we incubated live cells with each compound at a concentration of 100 μM, followed by labeling with FP-Cy5 (Fig. 5b). These results showed that even at high concentrations, Z27 only blocked FP labeling of FphE in live cells, whereas N34 blocked FP labeling of FphH, and W41 was unable to block FP labeling of any of the Fph enzymes. Notably, the clogP values calculated for Z27, N34, and W41 are 2.6, 2.1 and 1.4 (Table S2), respectively. This suggests that W41, having IC_50_ = 500nM, likely failed to bind FphE in live cells due to overall low cell permeability compared to Z27 and N34. Overall, the results for Z27 and N34 are consistent with the enzyme inhibition assay and suggest that they are likely valuable tools for selective inhibition of FphE and FphH.

To obtain further insight into the covalent binding mechanism of the boronic acid hits, we determined the co-crystal structures of FphE bound to Z27, N34 and W41, as well as a promiscuous borolane hit Q41, and the structure of N34 bound to FphH (Fig. 5c). Previously, we reported that FphE forms an unusual, interconnected homodimer in which the active site region is formed by residues from each of the two monomers^11^. Specifically, the active site triad is formed by Ser103 and Glu127 from one monomer and His257 from the other. The co-crystal structure of all boron containing ligands, either in the form of a boronic acid or a borolane warhead, showed a unique binding mode in the FphE active site, where Ser103 and His257 were both involved in making covalent bonds to the inhibitor boron atom to produce a tetrahedral adduct (Fig. 5c). This suggests that boron ligands bind through a mode which temporarily locks the two monomers and prevents dissociation of the dimer. For Z27, we also found one additional unreacted Z27 molecule at the edge of the active site that was aligned to form π-π interactions with two aromatic residues, Trp197 and Phe234 (Fig. 5d). This suggests that it may be possible to extend the Z27 fragment structure to occupy this additional binding site to improve both selectivity and potency for FphE. For N34, we observed a similar di-covalent binding mode as Z27 in the FphE active site however we did not observe the second molecule bound in the alternate binding site (Fig. 5e). This lack of a second molecule of N34 could explain the difference in potency observed between the two relatively similar boronic acid fragments.

For FphH, despite attempts to co-crystalize it with multiple hit compounds (X13, X13a, β-lactam, and imidazole-based hits) identified in the screening, we were only able to obtain a structure with N34 bound. This structure reveals that ligand binding to FphH introduces significant changes to the overall protein fold that influence crystal contacts, often preventing crystal growth. Indeed, the N34 bound FphH crystal structure forms a very different crystal form compared to the previously determined unbound structure^12^. In addition, the crystal interface in this new form is facilitated by two unreacted N34 molecules (Figure S6a). The N34 bound FphH structure shows a deep hydrophobic pocket occupied by the trifluoromethyl moiety of N34, and the boronic acid bound to the catalytic serine and histidine in the active site suggesting a strong interaction which likely contributes to its nanomolar inhibition potency (Fig. 5f). There are subtle changes in the active site pocket compared to the unbound state (Figure S6b) with some areas being narrower to facilitate interactions with the ligand, while others more open to give the ligand the necessary room for binding. More drastic changes are seen in the lid region of FphH with several helices folding differently in the N34 bound state (Figure S6b). Alignment of bound and unbound FphH results in a significant RMSD of 1.6 Å when using all Cα atoms. The lid or cap region is critical for many serine hydrolases such as FphH^12^, thus compounds such as N34 that are able to introduce changes in this region likely will result in potent inhibition.

### Selective inhibitors of each class affected bacterial growth and show antibiofilm activity

**Figure 6:**
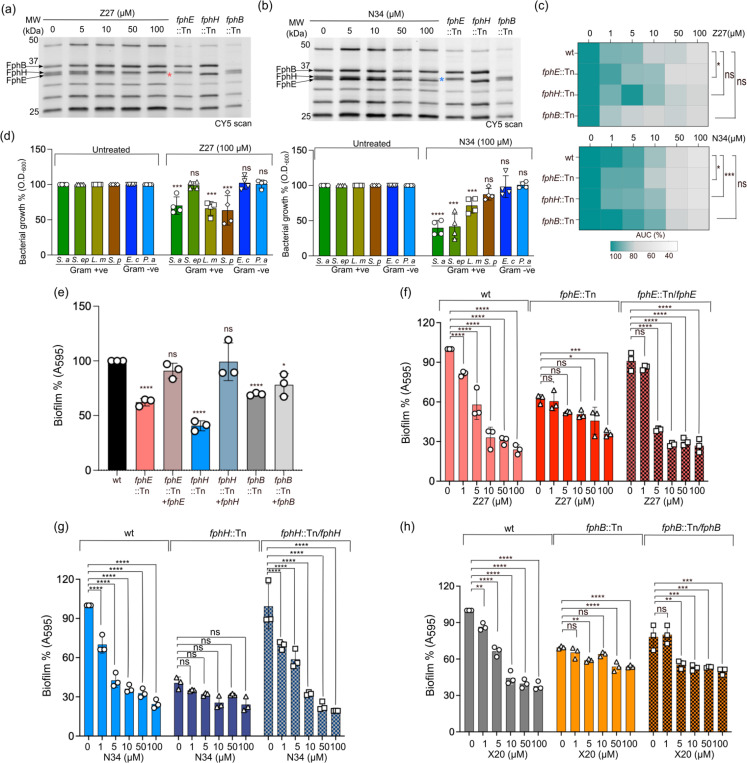
Boronic acid-based inhibitors show antibacterial and antibiofilm activity. Fluorescence SDS-PAGE gel images of live *S. aureus*USA300 WT cell preincubated for 120 min with the indicated concentrations of Z27 (a) and N34 (b) and then labeled with the broad-spectrum serine hydrolase FP probe. The individual transposon mutants of *fphB, fphH and fphE* are included to indicate the location of the three enzymes in the gel. All the cells were labeled with the clickable FP-alkyne, lysed and labelled with Cy5-azide and analyzed by SDS-PAGE followed by fluorescence scanning. (c) Heat maps derived from growth curves to compare the area under the curve (AUC) for *S. aureus wild type and S. aureus fph transposon mutants* after treatment with the indicated concentrations of Z27 or N34 (Generated from Figure S5d & e). *AUCs are normalized such that 100% represents the AUC for untreated* wild-type *S. aureus.* The graphs show mean ± standard deviation of data from n = 2 biological replicates (each as technical duplicates). *(d) Effects of* Z27 and N34 on the indicated bacteria. Gram-positive bacteria tested: *S. a*: *S. aureus, S. ep: S. epidermidis, L. m: Listeria monocytogenes, S. p: Streptococcus pyogenes.* Gram-negative bacteria tested *E. c: Escherichia coli (E. coli), P. a: Pseudomonas aeruginosa.* The data plotted are growth percent relative to the untreated control at the 10hr time point for each strain. The graphs show mean ± standard deviation of data from n = 2 biological replicates (each as technical duplicates). *(e)* Percentage biofilm formation measured by crystal violet staining for *S. aureus wild type, fph mutants, and complement strains. Results are normalized such that 100% represents the signal from WT S. aureus (f)* Percentage of biofilm inhibition by Z27 measured for wild-type *S. aureus, fphE mutant, and the corresponding fphE complement. Results are normalized such that 100% represents levels for untreated WT S. aureus (g)* Percentage of biofilm inhibition by N34 measured for wild-type *S. aureus, fphH mutant, and fphH complement. (h)* Percentage biofilm inhibition by X20 measured for wild-type *S. aureus, fphB mutant, and FphB complement. Results are normalized such that 100% represents untreated WT S. aureus.* Bars represent means ± standard deviation of data from n = 3 biological replicates. P values were determined using ordinary one-way ANOVA multiple comparisons. *P < 0.05, **P < 0.01, ***P < 0.001 and ****P < 0.0001.

Given that several of the boronic acid electrophile fragments showed high potency and selectivity against both recombinant Fph targets as well as the native proteins in *S. aureus* cells, we next wanted to apply these molecules for functional studies. We first measured the selectivity of Z27 and N34 in live cells. Pretreatment of cells with increasing doses of compounds from 5–100 μM showed that each was able to block the activity of the intended Fph target as measured by loss of labeling by the FP probe (Fig. 6a). We found that Z27 effectively blocked only FphE labeling and completely inhibited labeling at 50 μM concentrations and higher. N34 also showed a preference for its primary target, FphH, but also showed partial inhibition of FphE and other lower molecular weight Fph proteins when used at concentrations at or above 50 μM.

To further assess the selectivity of the boronic acid hits, we performed a similar competition for FP labeling in *Staphylococcus epidermidis* which is a related bacterium that is predicted to have an FphH homolog but not an FphE homolog^11,35^. None of the compounds showed competition of FP labeling for any of the protein targets in *S. epidermidis* (Figure S7j). This data further confirms that Z27 and N34 are not generically reactive and in fact have high specificity for the *S. aureus* FphE and FphH enzymes.

Given the overall selectivity and potency, we observed in labeling of live bacteria, we wanted to use the compounds to determine if inhibition of FphB, FphE and FphH had effects on bacterial growth and biofilm formation. For these studies we grew wild-type *S. aureus* and transposon mutants of *fphB, fphE*, and *fphH* in the presence of a range of concentrations of Z27 or N34 (Fig. 6c, Figure S5g–o). From the resulting growth curves, we calculated the area under the curve (AUC) as a metric to compare the overall growth effects of the two compounds (Fig. 6c, Figure S5d–e). None of the transposon mutants grew differently compared to wild type in the absence of the inhibitors, consistent with our prior studies and suggesting that none of these enzymes alone are essential for bacterial growth. However, in the presence of N34, which inhibits FphH > FphE, both the *fphE* and *fphH* mutants grew slower than wild type and caused a characteristic biphasic growth pattern at high concentrations of N34 (Figure S5g–h). The FphE selective inhibitor Z27 resulted in reduced growth for only the *fphE* mutant, likely due to its off target inhibition of FphH (Fig. 6c, Figure S5k). These results indicate that inhibiting both Fph proteins has a more pronounced impact on bacterial growth, consistent with potential overlapping functions of these two enzymes.

To confirm that our two boronic acid inhibitors do not have general toxic effects due to inhibition of targets other than the Fph proteins, we tested the compounds in several Gram-positive and Gram-negative bacteria. Our prior analysis of protein homology predicts that Fphs are generally less conserved in Gram-negative bacteria^11^. Among Gram-positive bacteria, *Listeria monocytogenes* shares the highest homology with *S. aureus* for FphE and FphH, while *S. epidermidis* has an FphH homolog but lacks FphE, and *Streptococcus pyogenes* does not have either an FphE or FphH homolog. Interestingly, we observed a similar growth inhibition for *L. monocytogenes* in the presence of Z27 and N34 suggesting that these compounds might target both enzymes in both species (Fig. 6d, Figure S7a–c). As expected, *S. epidermidis*, lacking FphE, was inhibited only by the FphH inhibitor N34, and no growth inhibition was observed for *S. pyogenes* for N34 (Figure S7d, S7g–h). In addition, no significant growth defect was observed for two Gram-negative bacteria (*Escherichia coli* and *Pseudomonas aeruginosa*) for either Z27 or N34 (Fig. 6g, Figure S7e–f). The absence of activity against these bacteria could also be due to the permeability barrier from the outer membrane. Overall, these results further suggest that the growth defects observed in *S. aureus* are unlikely to be due to off-target toxicity. Finally, to further rule out general toxic effects of the compounds we treated HEK293 cells with a range of concentrations of Z27 and N34 and observed no toxicity to the cells even after 48hr of incubation (CC_50_ > 200μM; Figure S7k).

Finally, we wanted to determine if our newly identified selective inhibitors could be used to assess the functional roles of the Fph proteins in biofilm formation. Using a standard crystal violet biofilm assay, we first assessed the impact of disruption of each of the FphE, FphH, and FphB genes on biofilm formation. We found that the *fphH* transposon mutant showed the most pronounced effects with more than 50% reduction in biofilm formation while *fphE* and *fphB* mutants showed a more modest 20–30% reduction in biofilm formation (Fig. 6e). These data suggest that all three enzymes likely play some role in biofilm formation. However, genetic disruption of protein expression can result in compensatory effects in the bacteria which make it difficult to assess the individual contributions of each enzyme to the biofilm formation process. Therefore, we tested the effect of chemical inhibition of FphB, FphE and FphH on biofilm formation for wild type, transposon mutants and rescue strains using the Z27, N34 and X20 inhibitors (Fig. 6f–h). This allowed us to confirm that the effects from the transposon insertional silencing of each gene could be restored by complementation of the native gene. The results from these studies demonstrated that all three compounds caused a reduction in biofilm formation in the wild type bacteria over the range of doses tested with the most pronounced effects observed for N34, consistent with the transposon data suggesting that FphH has the most significant role in this process of the three Fph enzymes. Furthermore, we again observed a large (greater than 50%) drop in biofilm formation in the FphH transposon mutant and this drop was not further increased by addition of even 100 μM concentrations of N34. Importantly, biofilm formation was restored to WT levels by complementation of the FphH protein in the transposon mutants and this also restored the dose-dependent inhibitory activity of N34 suggesting that the effects of the compound are mediated by inhibition of the intended target, FphH (Figure S8a). Similar analysis of the *fphB* and *fphE* transposon mutants demonstrated that loss of expression of each of these proteins resulted in only 35–40% inhibition of biofilm formation. The level of biofilm formation was further reduced by the inhibitor Z27 only at the highest concentrations used (50 and 100 μM), where it starts inhibiting FphH as shown by the dose-dependent inhibitory activity for the *fphE* transposon mutants (Figure S8b). For, X20 we observed significant dose dependent inhibition of biofilms for WT cells with 60% reduction of biofilms at the highest concentrations as compared with a 40% reduction for the *fphB* mutant, suggesting that X20 may be targeting other Fphs in the absence of FphB. We observed a similar level of reduction in biofilms for *fphE* and *fphH* transposon mutants as the *fphB* mutant (Figure S8c). Similarly, complementation of both FphB and FphE restored biofilm formation and the inhibitory activity of the compounds. These data suggest that at low concentrations, the two compounds primarily target the intended Fph targets and the enhanced effects at higher concentrations in the transposon mutants are likely due to off target inhibition of FphH or other Fph proteins. Thus, our data suggest that inhibition of FphH alone is sufficient to dramatically impact biofilm formation. However, to obtain the same level of inhibition of biofilm formation for FphB and FphE targets requires inhibition of additional Fph enzymes.

The strong antibiofilm activity of the boronic acid compounds argues for their possible use in combination therapy with antibiotics to treat staphylococcal infections. Therefore, we wanted to test if the N34 treatment impacts the susceptibility profile of the *S. aureus* to antibiotics. To test this, we pretreated bacteria with a sub-inhibitory concentration of N34 (20 μM) for 2 hours at 37°C followed by treatment with selected antibiotics. As shown in Table S5, all the antibiotics have similar half-effective maximal concentrations in both the DMSO- and N34-treated cells, indicating that N34 does not impact resistance or susceptibility profiles of the antibiotics.

## Discussion

In this study, we demonstrate that screening of a serine-focused electrophile fragment library identified selective inhibitors of three important serine hydrolases in *S. aureus* that have the ability to cleave highly similar substrates. Our screens produced lead molecules with not only high selectivity but also high potency without the need for significant additional medicinal chemistry efforts. These results are in contrast to traditional reversible binding fragment-based screening efforts for other targets where hits tend to bind with high micromolar or even millimolar affinities^36,37^. This discrepancy may be due both to the use of covalent fragments which require minimal surface area to form a productive interaction with the target and the fact that our serine hydrolases cleave relatively unbranched lipid substrates that can be easily mimicked with small, generally hydrophobic fragments found in these types of commercial libraries. Regardless, our results suggest that this approach and library composition may be highly fruitful for screening of other serine hydrolase targets, especially if those targets that process saturated lipid substrates.

Our screen identified the fluorosulfate (OSF) electrophile as an inhibitor of multiple Fph targets. Further analyses confirmed that these hits covalently target the active site serine of FphH. Typically, OSF electrophiles covalently label residues such as Lys, His, or Tyr^30^. In this study, the discovery of the saccharine-like active cyclic intermediate of X13 suggests that these types of molecules could be used as prodrugs for serine hydrolase inhibitors since the cyclization makes them likely to be serine specific and potentially less reactive than the OSF electrophiles. The ability of this specific class of OSF electrophiles to convert into saccharine-like compounds to provide selectively for targeting serine suggests a unique interaction mechanism that merits further investigation.

The sulfonyl fluoride-based hit identified for FphB (X20) is an irreversible covalent inhibitor and showed a significant inhibition of biofilm formation despite having minimal effect on planktonic growth. Moreover, X20 did not show any off-target effects in mammalian cells. This selective action can be beneficial because biofilm-associated infections are a major clinical challenge, particularly in chronic infections, medical implants, and catheter-related infections. An inhibitor that targets biofilm formation without killing planktonic cells may reduce the likelihood of resistance development, as it does not exert broad selection pressure on the bacteria.

Several approved boronic acid-based drugs are on the market, including vaborbactam and bortezomib, which target the catalytic serine in the enzyme active site of serine hydrolases by forming covalent, reversible interactions^38,39^. The unique mechanism, which involves the formation of metastable tetrahedral adducts, positions boronic acids as effective “serine traps”^40^. The success of boronic acid-based inhibitors as drugs highlights the potential of these inhibitors to serve as potent antibacterial leads^41,42^. In our screening, we identified two boronic acid-based inhibitors Z27 and N34 with nanomolar inhibition potency without any structural optimization, highlighting the inherent potency of boronic acid scaffolds in targeting serine hydrolases.

In this study, we also determined the co-crystal structures for Z27, W41, Q41 bound by FphE and N34 bound by both FphE and FphH. All the inhibitors bound Ser and His in the active site to produce a tetrahedral adduct. The co-crystal structures of FphE reveal a di-covalent binding mode, in which both Z27 and N34 temporarily lock the two monomers and stabilize the dimer. Moreover, an additional unreacted Z27 molecule binds near the active site pocket through π-π interactions, suggesting a potential route for chemical derivatization, which could enhance the potency or confer additional specificity to improve the efficacy of the hit compounds. Binding to FphH appears to facilitate major changes to the protein fold that may result in prevention of crystal growth for some ligands or facilitate new crystal forms, with N34 being an example of the later. This structure demonstrates that FphH has dynamic properties at the active site that is occupied by the ligand and also that ligands are able influence the critical lid (cap) region^12^. This structure also provides structural insights that could inform more robust inhibitor design against this important serine hydrolase.

Interestingly a fragment closely resembling Z27 has been reported^41,42^. This fragment, benzo[b]thiophene-2-boronic acid “BZBTH2B”, contains the boronic acid group directly adjacent to the sulfur molecule in the 5 membered ring, which is structurally different from Z27 (Benzo[b]thiophen-3-ylboronic acid). For the *E. coli* β-lactamase (AmpC), BZBTH2B showed a similar inhibition potency as Z27 for FphE (IC_50_ = 27nM)^42^. The tetrahedral configuration reported in this work reveals a di-covalent binding mode of boron to serine and histidine, which is a rare occurrence for serine hydrolases. The reports on similar di-covalent interactions for boronic acid-based inhibitor to the active site triad residues has only been demonstrated for serine proteases such as subtilisin or γ-chymotrypsin (PDB ID 4VGC)^43,44^.

Serine hydrolases often show overlapping or compensatory functions^46,47^. In a recent report, we showed the overlapping functions of serine hydrolases in *Plasmodium falciparum* and explored strategies to inhibit multiple targets simultaneously to improve parasite killing^15^. In this study, we show that Z27 and N34 selectively inhibit the activity of FphE and FphH in *S. aureus*. Here we show that simultaneously inhibiting both FphE and FphH with N34 can counteract the potential compensatory functions of these enzymes to reveal their roles in bacterial growth and biofilm formation. This study highlights the efficacy of boronic acid compounds in targeting multiple serine hydrolases in *S. aureus* and opens new directions for exploring novel boronic acid-based therapeutics for bacterial infections.

Our biofilm studies show that among the three Fphs tested, FphH plays the most essential role in biofilm formation. The transposon mutant and complementation experiments further supported this, as inhibiting FphH showed significant biofilm disruption. The other two Fphs (FphE and FphB) showed only a weak effect of biofilm formation, suggesting that inhibitors can enhance the effects at high concentrations likely because they can inhibit multiple Fph proteins. Our study supports the notion that the most effective strategy to block biofilm formation is to either inhibit multiple Fph proteins simultaneously or specifically inhibit FphH. Since FphH alone showed a substantial role in biofilm formation as well as cell growth, it stands out as a viable target for therapeutic agents that function as both antibiotics and anti-biofilm agents.

In summary, this study highlights the power of using HTS of a serine-focused fragment library to identify highly selective covalent inhibitors for serine hydrolases in *S. aureus*. In particular, we discovered new electrophile classes, including the fluorosulfate, sulfonyl fluoride and boronic acid-based inhibitors, and demonstrated that these compounds can be used to selectively target each of the screened serine hydrolases in *S. aureus*, FphB, FphE, and FphH, by covalently binding to the active site serine. These selective hits demonstrate that inhibition of FphH affects the growth of bacteria that express this Fph enzyme. We also showed that all the three serine hydrolases play some role in biofilm formation, highlighting the potential for Fph inhibitors to be used for preventing biofilm-associated infections. Overall, we present a systematic approach to identifying highly selective covalent inhibitors for further development of antibacterial agents to combat *S. aureus* infections, where biofilm formation plays a crucial role in pathogenesis.

## Material and Methods

### Protein expression and purification

The *fphE* and *fphH* genes from *S. aureus* USA300 were expressed in pet28a-(pETNKI-his-3C-LIC-kan) as previously described^11,12^. Briefly, an overnight culture of the transformed bacteria in LB (+ Kanamycin 50 μg/ml) was diluted 1:100 into 1 L of selection medium and grown at 37°C, 220 rpm. At OD600 0.7– 0.8 the protein expression was induced by addition of isopropyl β-D-1-thiogalactopyranoside (IPTG) (0.2 mM final concentration) and grown overnight at 18°C, 180 rpm. For purification, cells were harvested by centrifugation, transferred to 50 mL conical tubes, centrifuged again and bacterial pellets stored at − 80°C. Each pellet was thawed on ice, resuspended in 30 mL lysis buffer (20 mM Tris pH 8.0, 300 mM NaCl, 0.05mg/mL lysozyme) and lysed by sonication (3 X 1min: pulse ON 4 sec. pulse OFF 1 sec, 50% amplitude, Branson Sonifier) followed by centrifugation at 15,000 rpm in Avanti JA-17 rotor for 45 min. Both the proteins were first purified by Ni-NTA resin pre-equilibrate affinity chromatography using equilibration buffer (50 mM Tris pH 8.0, 300 mM NaCl, 5 mM imidazole, 10% glycerol). The His6-tagged proteins were eluted in 50 mM Tris pH 8.0, 300 mM NaCl, 300 mM imidazole, 10% glycerol buffer. Recombinant FphE (rFphE) and Recombinant FphH (rFphH) were further purified by size exclusion chromatography using S200 10/300 column (EMD Millipore, Billerica, MA) 20 mM HEPES pH 7.0 and 100 mM NaCl). Finally, both purified rFphE and rFphH were concentrated and flash frozen for long-term storage at − 80°C. The concentration of purified rFphE and rFphH was determined by Bradford assay.

-term storage at − 80°C. The concentration of purified protein was determined by Bradford assay.

### Complementation of fph genes

The Sequence Ligation Independent Cloning (SLIC) method utilized Velocity polymerase (Qiagen), which generates blunt ends. Open reading frames of the Fph genes were cloned from the genomic DNA of the *S. aureus* USA300 LAC strain using the primers listed in Table S4. The pALC plasmid was cloned using Velocity polymerase and digested with DpnI at 37°C for 2hr. After purifying the pALC and gene template were treated with T4 DNA polymerase. The plasmid and insert were mixed and subjected to thermal cycling at 65°C for 10 minutes, gradually decreasing the temperature from 65°C to 25°C, with a 1-minute hold at each degree. The annealed products were pelleted, purified using Pellet Paint (Millipore), and resuspended in nuclease-free water. The purified product was transformed into electrocompetent *E. coli* (IM01B) via electroporation at 1.8 kV, 25 μF, and 200 Ohms. The transformants were resuspended in LB medium and incubated for one hour at 37°C. After incubation, the transformants were spread onto LB agar containing chloramphenicol and incubated at 37°C for 18 to 24hr. To select for transformants carrying the recombinant plasmid, single colonies were isolated and verified using PCR and DNA sequencing. Correct recombinant plasmids were then transformed into the *S. aureus* transposon mutants and selected on Tryptic Soy Agar plates with chloramphenicol. The presence of the recombinant plasmids in the *S. aureus* strains was confirmed using PCR and sequencing.

### FphE and FphH ligand bound crystallization

Production of *S. aureus* USA300 FphE^11^ and FphH^12^ for crystallography has previously been described in detail. In all cases, the N-terminal purification tag was cleaved off with a 3C protease, with residues GPG remaining at the N-terminus of the otherwise full-length FphE (Uniprot ID Q2FDS6) and FphH (A0A0H2XJL0) proteins. Protein concentrations for crystallography were determined by Absorbance at 280nm and calculated extinction coefficients. For FphE-Z27 co-crystallization, 20 μL of 25.0 mg/mL FphE (10 mM HEPES pH 7.5, 100 mM NaCl) were mixed with 3 μL of Z27 (50 mM in DMSO) and incubated at 4°C overnight. Next, 0.3 μL FphE-Z27 solution was mixed with 0.15 μL of reservoir solution. Sitting drop reservoir contained 25 μL of 180 mM Calcium acetate, 100 mM MES pH 6.5, 22.5% PEG 2000 MME. Crystal was frozen in a solution of ~ 25% glycerol, 75% reservoir. For FphE-N34 co-crystallization, 13 μL of 19 mg/mL FphE (10 mM HEPES pH 7.5, 100 mM NaCl) were mixed with 5 μL of N34 (50 mM in DMSO) and incubated at 18°C overnight. Next, 0.15 μL FphE-N34 solution was mixed with 0.3 μL of reservoir solution. Sitting drop reservoir contained 25 μL of 180 mM Magnesium chloride, 100 mM MES pH 6.5, 22.5% w/v PEG 2000 MME. Crystal was frozen in a solution of ~ 25% glycerol, 75% reservoir. For FphE-W41 co-crystallization, 20 μL of 25.0 mg/mL FphE (10 mM HEPES pH 7.6, 100 mM NaCl) were mixed with 3 μL of W41 (50 mM in DMSO) and incubated at 4°C overnight. Next, 0.15 μL FphE-W41 solution was mixed with 0.3 μL of reservoir solution. Sitting drop reservoir contained 25 μL of 180 mM Magnesium chloride, 100 mM Tris pH 8.5, 22.5% PEG 2000 MME. Crystal was frozen in a solution of ~ 25% ethylene glycol, 75% reservoir. For FphE-Q41 co-crystallization, 13 μL of 19.0 mg/mL FphE (10 mM HEPES pH 7.5, 100 mM NaCl) were mixed with 5 μL Q41 (50 mM in DMSO) and incubated at 18°C overnight. Next, 0.3 μL FphE-Q41 solution was mixed with 0.15 μL of reservoir solution. Sitting drop reservoir contained 25 μL of 180 mM Calcium acetate, 100 mM Tris pH 8.5, 22.5% PEG 2000 MME. Crystal was frozen in a solution of ~ 25% ethylene glycol, 75% reservoir. For FphH-N34 co-crystallization, 13 μL of 10 mg/mL FphH (10 mM HEPES pH 7.5, 100 mM NaCl) were mixed with 5 μL N34 (50 mM in DMSO) and incubated at room temperature (~ 22°C) for ~ 1 hour. Next, 0.2 μL FphH-N34 were mixed with 0.2 μL of reservoir solution. Sitting drop reservoir contained 180 mM Calcium acetate hydrate, 100 mM Tris pH 7.5, 9% w/v PEG 8000 and 9% w/v PEG 1000. Crystal was frozen in a solution of ~ 25% ethylene glycol, 75% reservoir.

### FphE and FphH ligand bound data collection, processing, refinement, deposition, and analysis

X-ray diffraction data were collected at the Australian synchrotron MX1^49^ and MX2^50^ beamlines. Datasets were processed with XDS^51^, merging and scaling were performed using AIMLESS^52^. Phases were solved with Phenix Phaser^53^ molecular replacement using previously determined FphE^11^ and FphH^12^ structures. Model building and refinement were conducted in COOT^54^ and Phenix^55^. The final structures were deposited to the worldwide protein databank^56^, see TableS6 and S7 for PDB IDs and dataset statistics. Structure figures, analysis and alignments were created with UCSF Chimera^57^.

### Fluorogenic Substrate Enzyme Activity Assay

Fluorogenic substrate assays were performed as previously described^9,11,12^. Briefly, In a 384-well plate, 50 μM 4-methylumbelliferone (4-MU)-conjugated substrates were combined with recombinant 100 nM FphB, 0.5 nM FphE and 5 nM FphH protein in the assay buffer (0.02%TritonX-100 in 1X PBS) and fluorescence (λex=365nm and λem=455nm) was measured at 30°C in 1 min intervals on a Cytation 3 imaging reader (BioTek, Winooski, VT, USA) for 60 min. Turnover rates in the linear phase of the reaction (10–20 min) were calculated using GraphPad prism 10.0 software as RFU/min. Rates were normalized by subtracting background hydrolysis rates measured for each substrate in reaction buffer in the absence of protein.

### High throughput screening

For the primary screening with the Enamine Serine Focused Covalent Fragments Library, a Beckman Echo 655 acoustic liquid handler was used to transfer 50 nL from source plates containing 10 mM DMSO stocks into assay plates for the final concentration of 50 μM for the screening. For the screening, 8 μL of enzyme solution was pipetted into the assay plates containing 50 nL compound and allowed to incubate for one hour at room temperature. Fluorescence intensities were measured after adding 2 μL 4MU substrate solution for each Fph proteins using an Agilent Cytation 3 Multi-Mode Reader (${\text{λ}}_{\text{ex}}=365\;\text{nm}$ and ${\text{λ}}_{\text{em}}=455\;\text{nm}$). For four-point dose response plates, 2.5, 10, 25, 50 nL droplets were transferred to get 0.8, 10, 25, 50 μM compound concentrations in the 384 well plates again using an Echo acoustic liquid handler as above. To determine the IC_50_ of the top hits (X13, X13a, X20, D17, L17, Z27, N34, W41, Q41, AF22, S31, T09, H08, AF26, Z08, and Y05) varied concentrations (0.01–50 μM) were preincubated with 100 nM FphB, 0.5 nM FphE and 5 nM FphH at room temperature for 1hr, and enzyme activity was measured by monitoring the change in fluorescence intensity after adding respective substrates for 1hr by using the Cytation 3 Multi-Mode Reader (${\text{λ}}_{\text{ex}}=365\;\text{nm}$ and ${\text{λ}}_{\text{em}}=455\;\text{nm}$). Calculations were done with GraphPad Prism 10.0 software. Sigmoidal curves were fitted to the data using the following dose– response equation:

$$y=A1+(A2-A1)/\left(1+\left(\frac{x}{IC50}\right)\right)$$

(x, inhibitor concentration; y, percent activity of the reaction; A1, 100%, y value at the top plateau; A2, 0%, y value at the bottom plateau; used standard slope of −1.0, Hill coefficient). IC50 values were derived from the fitted curve.

### Competition of inhibitors for activity-based recombinant protein labeling

For activity-based protein labeling experiments, 500 nM of rFphB, rFphE and rFphH in 1X PBS and 0.1% Triton–X were preincubated with different concentrations of inhibitors or DMSO for 2hr at room temperature, followed by adding 300 nM of fluorophosphonate (FP) alkyne and incubated for 30 min at room temperature. Next, 20 μL of the reaction volume were mixed with 2.16 μL of freshly prepared click mix (0.5 μL of 50 mM CuSO_4_ in H_2_O, 1.16 μL of 100 mM BTTAA in DMSO, and 0.5 μL of N_3_-Cy5 in DMSO) and 1.16 μL of 300 mM sodium ascorbate solution in H_2_O. Samples were incubated at 37°C for 30 min, boiled in SDS loading buffer and analyzed by SDS–PAGE. Cy5 fluorescence was imaged using the Cy5 channel on a Typhoon 9410 Imager (Amersham Biosciences).

### Competition of inhibitors for activity-based protein profiling in live cells

One colony of each of *S. aureus* wild-type and transposon mutants *fphB*::Tn, *fphE*::Tn and *fphH*::Tn picked from growth on a Tryptic soya agar plate were inoculated in 5 mL of Tryptic soya broth (TSB). The overnight bacterial cultures were spun down at 5,000 g for 10 min and pellet was collected. The pellets were washed twice in 1X PBS buffer and resuspended in the same buffer. Cells were first preincubated with the indicated concentrations of inhibitors for 2hr at 37°C. The cells were washed twice with 1X PBS buffer. The resuspended pellets were incubated with Fluorophosphonate-alkyne (FP-alkyne) for 30 min at 37°C. Cells were washed again twice with 1X PBS buffer and resuspended in with 1X PBS and 0.1% Triton–X and lysed by beat beating at 4°C. Next, lysed cells (20 μL) were mixed with 2.16 μL of freshly prepared click mix (0.5 μL of 50 mM CuSO_4_ in H_2_O, 1.16 μL of 100 mM BTTAA in DMSO, and 0.5 μL of N_3_-Cy5 in DMSO) and 1.16 μL of 300 mM sodium ascorbate solution in H_2_O and incubated at 37 °C for 30 min. Next step, 20 μL of 4X SDS loading buffer was added in each sample and boiled at 100 °C for 10 min. The processed samples were allowed to cool and analyzed by SDS-PAGE (12%) running at 120 V in an electrophoresis chamber. Cy5 fluorescence was imaged using the Cy5 channel on a Typhoon 9410 Imager (Amersham Biosciences).

### LC-MS analysis of native protein labeling with inhibitors

Recombinant FphH (1 μM) in 1X PBS was incubated with 10 μM of X13, X13a, MH24, and MH33 or DMSO for 60 min at room temperature. Recombinant FphB (1 μM) in 1X PBS was incubated with 10 μM of X20. Next, 10 μL of the labeled sample was injected and analyzed by LC-MS. For LC-MS conditions, 95% solvent A (0.1% formic acid in LC-MS grade water), 5% solvent B (0.1% formic acid in LC-MS grade acetonitrile) at 0.6 mL/min was used for 2 min to remove excess of salts into waste before a linear 6-minute gradient from 80% solvent A (20% solvent B) to 20% solvent A (80% solvent B), followed by a one-minute 95% solvent A (5% solvent B). Protein labeling efficiency was quantified by UV. The acquired mass of protein was deconvoluted using the deconvolution software in Agilent Bioanalysis Software package.

### LC-MS/MS analysis of X13 and X13a adduct on FphH

Recombinant FphH protein in PBS (1 μM; 13.5 μL) was incubated with 5 μM X13, 5 μM X13a, or DMSO (0.5 μL of 50x stock in DMSO) for one hour at room temperature, Proteinase K (NEB, 1 μL of 50x stock in PBS for final 1:100 w/w ratio to FphH) was then added and incubated at 24°C for exactly one min. Samples were then boiled for 10 min at 95°C to inactivate proteinase K. Proteins were then denatured in 6 M urea in 50 mM NH_4_HCO_3_, reduced with 10 mM neutralized TCEP (200 mM fresh stock in water) for 30 minutes at room temperature and alkylated with 25 mM iodoacetamide (400 mM fresh stock in water) for 30 minutes at room temperature in the dark. Samples were diluted to 2 M urea with 50 mM NH_4_HCO_3_, and digested with trypsin (Thermo Scientific, 1 μL of 0.5 μg/μL) in the presence of 1 mM CaCl_2_ (100x stock in water). The digestion was performed for 5 h at 37°C. Samples were acidified with 1 vol. of isopropanol 1% TFA and desalted using styrene divinylbenzene reverse-phase sulfonate (SDB-RPS) StageTips as described previously^58^. Briefly, samples were loaded on a 200 μL StageTip containing two SDB-RPS disks and centrifuged at 1500 x g for 8 min. This was repeated until all the sample was loaded on the StageTip. StageTips were washed three times with 200 μL of isopropanol 1% TFA at 1500 x g for 8 min, then eluted with 100 μL of 80% MeCN, 19% water, and 1% ammonia and dried. Peptides were resuspended in water with 0.1% formic acid (FA) and analyzed using nanoElute 2 coupled to a TimsTOF HT mass spectrometer (Bruker Daltonics). The chromatography column consisted of a 25 cm long, 150 μm i.d. microcapillary packed with 1.5 μm C18 particles (Bruker Daltonics/Pepsep) and was capped by a 20 μm emitter (Bruker Daltonics). LC solvents were 0.1% FA in H_2_O (Buffer A) and 0.1% FA in MeCN (Buffer B). Peptides were eluted into the MS at a flow rate of 500 nL/min. over a 15 min. linear-gradient (5–35% Buffer B) at 50°C. Data was acquired via dda-PASEF using 10 PASEF ramps per one MS1 scan and fragmented ions with charges 0 to 5. The method covered m/z 100–1700 and scan range between 0.6 to 1.6 1/K0. Ramp time was set to 100 ms and total cycle lasted 1.17 s. Collision energy was set as a linear increase from 20 eV at 1/K0 = 0.6 to 59 eV at 1/K0 = 1.6.

### FragePipe analysis

The MS data was analyzed with FragPipe (V21)^59,60^ and searched against the Human proteome with the addition of the FASTA sequence of FphH and a common list of contaminants (generated in FragPipe). Peptide and fragment mass tolerance was set to 20 ppm. The minimum peptide length was set to 6 amino acids with a mass range of 500 to 5000 Da. Trypsin (KR) and Proteinase K (FYWGAVLMI) were used as proteases with up to 2 and 10 missed cleavages, respectively. Carbamidomethylation of cysteine was set as fixed modification and oxidation of methionine, N-terminal acetylation, X13 (+ 290.0361 Da), and X13a (+ 276.0205 Da) on DEHKSTYR as variable modifications. PSM validation was performed with Peptide Prophet with the --decoyprobs --ppm --accmass --nonparam –expectscore parameters. Quantification was performed using IonQuant.

### Bacterial growth inhibition assays

Bacteria were streaked from glycerol stocks onto agar plates and grown overnight at 37°C. A single colony was inoculated in either Tryptic Soy Broth (TSB; Sigma-Aldrich, St. Louis, MO; used for most species) or Bacto Brain Heart Infusion (BHI; Becton, Dickinson, and Company, Franklin Lakes, NJ; used for *L. monocytogenes* and *S. pyogenes*) and grown at 37°C shaking until mid-exponential phase (OD_600_ = 0.5), then diluted 1:10 in cation-adjusted Mueller Hinton Broth (MHB2, Millipore Sigma, Bedford, MA; used for most species) or BHI (for *L. monocytogenes* and *S. pyogenes*). 15 μl of this dilution was added to 135 μl of MHB2 or BHI in a flat-bottom clear 96-well plate for a starting inoculum of ~ 5 × 10^5^ CFU/ml.

For bacterial growth inhibition assay, wild-type *S. aureus* and transposon mutants (*fphB*::Tn, *fphE*::Tn and *fphH*::Tn) were grown with compounds at final concentrations ranging from 1–100 μM and compared to growth in media with an equivalent percentage of DMSO. Each condition was plated as technical duplicates. The plate was incubated at 37°C with shaking every 15 minutes followed by measurement of OD_600_ for 14 hr. All growth curves were repeated at least twice and analyzed in GraphPad Prism 10.0 software. For bacterial resistance analysis, overnight culture of *S. aureus* Newman was diluted to an OD_600_ of 0.01 in MHB. This was followed by pretreatment with 20 μM N34 or equivalent DMSO volume as control. The pretreatment was allowed to proceed for 3 hr before initiating treatment with different concentrations of the test antibiotics (Table S5). The cultures were incubated overnight at 37°C, 5% CO2 before their optical density was read. The EC_50_ of the antibiotics against the bacteria in the presence or absence of N34 were determined by fitting the normalized data into a four-parameter logistic model in GraphPad Prism 10.0 software.

### Biofilm Inhibition Assay

Overnight cultures of *S. aureus* (wild-type, transposon mutants and complemented) strains (Table S3) grown in TSB, were subcultured on fresh TSB and grown to OD_600_ of 0.5. Biofilms were cultured in 96 well plates by transferring 1 μL of the culture to 200 μL of TSB plus 1% glucose with or without the inhibitors and incubated for 16 hours at 37°C without agitation. The inhibitor concentrations ranged from 1, 5, 10, 50 and 100 μM final concentration. Equal volume of DMSO was added as control. The wells were washed to remove the planktonic cells and the plates air-dried before staining with 200μL of 0.1% w/v crystal violet for 10 minutes at room temperature. The wells were washed to remove excess dye and blotted to dry. Each well was filled with 200μL of DMSO for 10 minutes at room temperature to dissolve the dye. The absorbance was measured at 590 nm using Spark multimode microplate reader (TECAN). The data represents n = 3 independent experiments and are expressed as a percentage of the uninhibited wild-type bacteria. All the data are normalized such that 100% represent untreated WT *S. aureus*. P values were determined using ordinary one-way ANOVA multiple comparisons GraphPad Prism 10.0 software. *P < 0.05, **P < 0.01, ***P < 0.001 and ****P < 0.0001.

### Cytotoxicity Assay

HFF cells were seeded into a 96-well side black-clear bottom plate at a density of 20,000 cells per well in 50 μL of complete growth medium (DMEM + 10% FAS) and allowed to adhere for 3hr at 37°C in a humidified incubator with 5% CO. Following adhesion, 50 μL of complete growth medium containing DMSO or various concentrations of the indicated compounds (0–200 μM, with a maximum DMSO concentration of 1%) was added to each well, bringing the total volume to 100 μL. The plate was incubated for an additional 24hr at 37°C. After incubation, 20 μL of CellTiter-Blue Reagent was added to each well, and the plate was returned to the incubator for a further 6 hr. Fluorescence was measured using a plate reader at an excitation wavelength of 560 nm and an emission wavelength of 590 nm. Fluorescence values were normalized to the DMSO-treated wells (100% viability) and to wells treated with 0.1% SDS, used as a negative control to represent 0% viability. The experiment was performed in biological triplicate and analyzed in GraphPad Prism 10.0 software.

## Supplementary Material

Supplement 1
