# Supplementary material for: Covalent-fragment screening identifies selective inhibitors of multiple Staphylococcus aureus serine hydrolases important for growth and biofilm formation
Source: Res Sq. 2024 Dec 13:rs.3.rs-5494070. Preprint. [Version 1] doi: 10.21203/rs.3.rs-5494070/v1 (PMC11661381; doi:10.21203/rs.3.rs-5494070/v1)
Supplement: Supplement 1 [file NIHPPRS5494070V1-supplement-1.pdf]

Supplementary Files

This is a list of supplementary files associated with this preprint. Click to download.

- UpadhyayFragmentsSupMat.docx
